# Supplementary material for: MARGINAL: An Automatic Classification of Variants in BRCA1 and BRCA2 Genes Using a Machine Learning Model
Source: Biomolecules. 2022 Oct 24;12(11):1552. doi: 10.3390/biom12111552 (PMC9687470; doi:10.3390/biom12111552)
Supplement: Supplementary file 1 [file biomolecules-12-01552-s001.zip › biomolecules-1886768-supplementary.pdf]

biomolecules

MDPI

Article

MARGINAL: An Automatic Classification of Variants in *BRCA1* and *BRCA2* Genes Using a Machine Learning Model

Vasiliki Karalidou [1,*], Despoina Kalfakakou [2], Athanasios Papathanasiou [2], Florentia Fostira [2] and George K. Matsopoulos [1]

[1] School of Electrical and Computer Engineering, National Technical University of Athens, 15780 Athens, Greece
[2] Molecular Diagnostics Laboratory, INRaSTES, National Center for Scientific Research NCSR Demokritos, 15341 Athens, Greece
* Correspondence: vasokaralidou@yahoo.gr

Supplementary Materials

Table S1. Misclassified variants predicted by eight ML algorithms for classifier 1 based on the test set provided by CanVaS database.

ML Algorithms	Variant Location
LR	13:g.32893172T>C
	13:g.32893344A>G
	13:g.32899271T>A
	13:g.32903606_32903607del
	13:g.32911193C>G
	13:g.32911300_32911303del
	13:g.32911436A>C
	13:g.32911614G>C
	13:g.32911628G>A
LDA	13:g.32893172T>C
	13:g.32893344A>G
	13:g.32899271T>A
	13:g.32903606_32903607del
	13:g.32911193C>G
	13:g.32911300_32911303del
	13:g.32911436A>C
	13:g.32911614G>C
	13:g.32911628G>A

KNN	13:g.32893344A>G
	13:g.32899271T>A
	13:g.32903606_32903607del
	13:g.32906834C>G
	13:g.32906957C>T
	13:g.32911193C>G
	13:g.32911300_32911303del
	13:g.32911436A>C
	13:g.32911565A>G
	13:g.32911614G>C
	13:g.32911628G>A
CART	13:g.32893344A>G
	13:g.32899271T>A
	13:g.32903606_32903607del
	13:g.32907401G>C
	13:g.32911193C>G
	13:g.32911300_32911303del
	13:g.32911436A>C
	13:g.32911614G>C
	13:g.32911628G>A
BernoulliNB	13:g.32893344A>G
	13:g.32893390A>C
	13:g.32899271T>A
	13:g.32907147C>A
	13:g.32907502_32907503insA
	13:g.32911193C>G
	13:g.32911300_32911303del
	13:g.32911628G>A
	13:g.32893344A>G
	13:g.32899271T>A
	13:g.32903606_32903607del
	13:g.32906834C>G

SVM	13:g.32906957C>T
	13:g.32907502_32907503insA
	13:g.32911193C>G
	13:g.32911300_32911303del
	13:g.32911436A>C
	13:g.32911628G>A
	13:g.32893344A>G
	13:g.32899271T>A
	13:g.32903606_32903607del
RF	13:g.32911193C>G
	13:g.32911300_32911303del
	13:g.32911436A>C
	13:g.32911614G>C
	13:g.32911628G>A
	13:g.32893344A>G
	13:g.32899271T>A
	13:g.32903606_32903607del
MLP	13:g.32911193C>G
	13:g.32911300_32911303del
	13:g.32911436A>C
	13:g.32911614G>C
	13:g.32911628G>A

Table S2. Misclassified variants predicted by eight ML algorithms for classifier 2 based on the test set provided by CanVaS database.

ML Algorithms	Variant Location
LR	13:g.32906732C>T
LDA	13:g.32906732C>T
	13:g.32907020_32907021del
KNN	13:g.32906732C>T
	13:g.32907020_32907021del
CART	13:g.32893474A>G
	13:g.32906732C>T
BernoulliNB	13:g.32906732C>T
SVM	13:g.32906732C>T
	13:g.32907020_32907021del
RF	13:g.32893474A>G
	13:g.32906732C>T
MLP	13:g.32893474A>G
	13:g.32906732C>T

Table S3. The total number of final labels predicted by the best ML algorithms (e.g., RF for classifier 1 and LR for classifier 2) for each class based on CanVaS database and the total number of labels provided by ClinVar database.

Classifier	Classes	Total Final Predicted Labels	Total ClinVar Labels
1	Other variants	64	53
	VUS	36	47
2	B/LB	37	27
	P/LP	37	25
	VUS	-	22
